# Maturation of pluripotent stem cell derived cardiomyocytes: The new challenge

**DOI:** 10.21542/gcsp.2016.6

**Published:** 2016-03-31

**Authors:** Idil Aigha, Christophe Raynaud

**Affiliations:** Qatar Cardiovascular Research Center, Qatar Foundation, Education City, Doha, Qatar

## Abstract

Stem cell therapy appears to be a promising area of research for cardiac regeneration following ischemic heart failure. However, *in vitro* differentiation of cardiomyocytes from pluripotent stem cells, or directly from somatic cells, leads to generation of “immature” cardiomyocytes that differ from their adult counterparts in various ways. This immaturity triggers some challenges for their potential clinical use, and multiple techniques reviewed here have been developed for *in vitro* maturation of those cells. Nevertheless, full maturity of cardiomyocytes remains elusive and will remain the main challenge for stem cell therapy in the near future.

## Introduction

Heart disease continues to be the leading cause of deaths in the western world. In a statistical report from 2010, it was found that more than 2000 Americans die each day from cardiovascular diseases (CVD), equivalent to 1 death every 40 seconds^1^. Coronary heart disease is the most common form of CVD and most often results from a buildup of plaque which leads to a narrowing of the blood vessels. This condition can lead to the loss of viable myocardium and progress into heart failure^2^. Despite advances in medicine, heart transplantations remain the most effective treatment strategy for massive heart failure, which is restricted by the limited number of donors and immunological problems^3^.

Being terminally differentiated, postnatal or adult cardiomyocytes (CMs) have a limited capacity to regenerate and multiply, which make them insufficient for cell death compensation^4^. The generation of CMs *in vitro* – either from human pluripotent stem cells (hPSCs) or reprogrammed somatic cells – is believed to be a promising new therapeutic strategy for heart failure^5^. However, even if promising in animal models, this approach often results in only partial restoration of the cardiac contractile function, which is most likely due to the less mature phenotype of the injected cells compared to adult CMs.

CMs derived from PSCs, or even somatic cells, are more closely related to fetal CMs in their structure, proliferation rate, metabolism and electrophysiology than adult mature CMs^6^. Long studied in mice, a recent study tested this approach by engrafting human embryonic stem cells derived cardiomyocytes (hESCs-CMs) in non-human primates with induced myocardial infarction. They demonstrated that this approach could successfully re-muscularized most of the infarcts in the tested animals and that the new tissue could be re-vascularized.

However due to the incomplete maturation of the hESCs-CMs and electrical reentry points in the newly engrafted tissues, sever arrhythmias were observed in these animals^7^. This study demonstrated that the transplanted hPSCs-CMs could partially improve the cardiac function but it highlighted the need to develop techniques to obtain more mature phenotype of CMs before implantation and ensure better electrical coupling. In this review we will discuss the level of maturity of hPSC-CMs, their difference from adult CMs and the different methods developed so far to influence the maturation of hPSC-CMs.

## From cardiomyogenesis to PSCs cardiac differentiation

**Figure 1.**
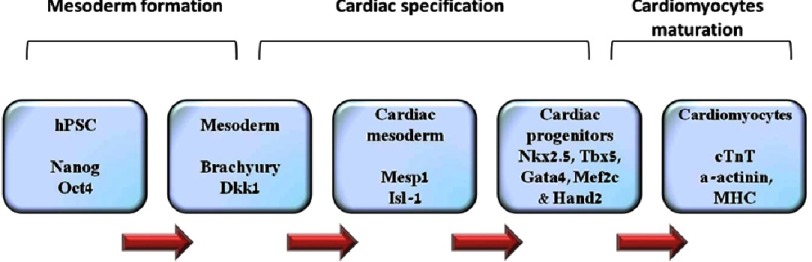
Schematic of current knowledge of stepwise differentiation of hPSCs into cardiomyocytes with involved transcription factors. Progression of cardiomyocytes differentiation from human pluripotent stem cells (hPSCs) and the factors involved in the different stages (pluripotent, mesoderm, cardiac mesoderm, cardiac progenitor and finally terminally differentiated cardiomyocytes).

PSCs differentiation into CM is achieved by mimicking the natural cardiomyogenesis process during embryonic development^8^. Several signaling pathways, growth factors and transcription factors have essential roles in the differentiation into CMs^9^. Cells proceed from pluripotent state, through mesoderm, cardiac mesoderm and cardiac specification, under the influence of successive cytokine stimulation, such as FGF2, Nodal, BMP4 and DKK1^10^. These cytokines, in turn, trigger the expression of transcription factors such as MESP1, Gata4, Hand2, MyoCD, Nkx2.5^11^ (Figure 1).

**Figure 2.**
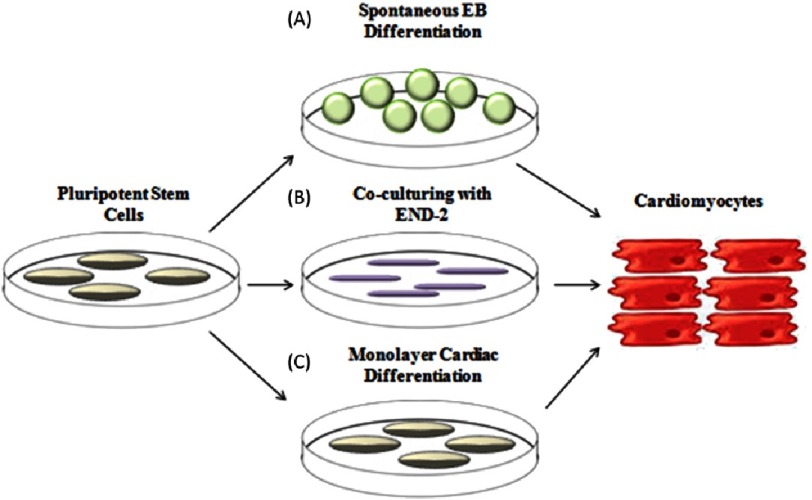
Schematic of different culture approach for cardiac differentiation. Three different methods used for cardiac differentiation of hPSCs. (A) Cardiac differentiation of embryoid bodies. (B) Co-culturing of hPSCs with mouse visceral endodermal-like (END-2). (C) Monolayer-based cardiac differentiation.

The precise cocktail of cytokines and their time and duration of treatment have been optimized by numerous groups. Similarly, various culture conditions (monolayer-based, embryonic body-based, or even by means of co-culture) were tested and previously reviewed by Burridge et al. (Figure 2). Most methods using cytokines rarely exceeded 20% of efficient CM differentiation^12^. In addition, these protocols had to be refined for each specific PSC line.

Recently, the use of small molecules, instead of cytokines, was shown to induce differentiation in 80–90% of PSCs^13,14^. This exciting breakthrough reduced the need for CM enrichment and limits the risk of injection of undefined cells. However, in clinical applications, purified CMs would be needed.

## Direct reprogramming of somatic cells into cardiomyocytes

First demonstrated in mouse models in 2010^15^ and recently in humans^16–18^, direct reprogramming of human somatic cells (mainly fibroblasts) into CMs (without passing by the pluripotent stage) is an alternative approach. By transduction with retroviral vectors carrying cardiac specific transcription factors such as GATA4, Mef2c, Tbx5, Mesp1, Hand2 and MyoCD, human somatic cells can be reprogrammed into what was referred as “induced cardiomyocytes” (iCMs). The CMs produced rarely exhibit spontaneous contraction, but express cardiac specific genes such as *TNNT2* (sarcomeric protein). However, the reprogramming of fibroblast into CMs is inefficient, often results in only partial reprogramming, and a long culture time leads to a loss of contractility of the reprogrammed cells. Furthermore, the global gene expression of the reprogrammed cells showed even more immature phenotype than PSCs-CMs^18^.

## Structural and functional characteristics difference between adult mature and PSCs derived cardiomyocytes

### Morphology

**Figure 3.**
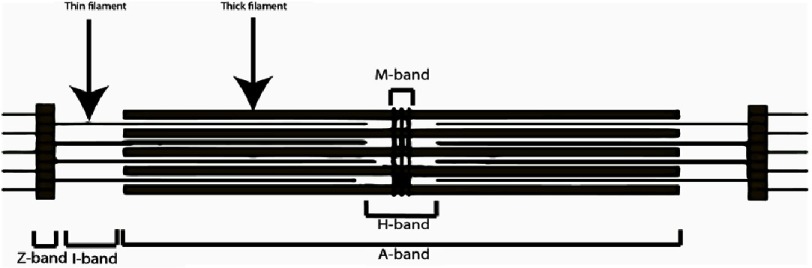
Schematic representation of the different bands observed in light microscopy of cardiomyocytes sarcomeric proteins. The A band appear dark as it is consisted of the thick and thin filaments while the thin filaments are known as I bands. The Z-band (where the actin filaments extend across) lies in the centre of I-band. The H-zone is in the center of A-band and bisected by a dark central line called the M-line.

Human adult CMs have a large rod-shaped phenotype^19^, and most of the cells are bi- or multi-nucleated^20^. They form myofibers by attaching to adjacent myocytes. Myofibers are bundled tubes divided into contracting units known as sacromeres, which in turn express contractile proteins such as actin and myosin^21^. Sacromeres are divided into sub-regions, or bands, which are named based on their light or dark appearance through the light microscope (Z-, A-, I-, H- and M - bands) (Figure 3).

In the Z-line regions, there are deep invaginations of the plasma membrane of the surface sarcolemma, known as transverse tubules (t-tubules) ^22^. T-tubules play an important role in the excitation-contraction coupling, by allowing the promotion of action potential into the cell interior, which in turn leads to the intracellular transient rise of Ca^2+^ concentrations^23^.

They also provide proximity between the sarcoplasmic reticulum (SR) (the main store for intracellular Ca^2+^) and the excitable cell membrane. The t-tubule membrane is facing the SR membrane, and at this junction the L-type Ca^2+^ channels are opposed to the SR Ca^2+^ release channels, known as ryanodine receptors (RyRs)^23,24^. Ca^2+^ is transferred following its release by sarco/endoplasmic reticulum Ca^2+^ ATPase (SERCA), from the cytoplasm into the lumen of SR. The appearance of t-tubules is considered to be one of the main hallmarks of mature cardiac development^25^.

**Figure 4.**
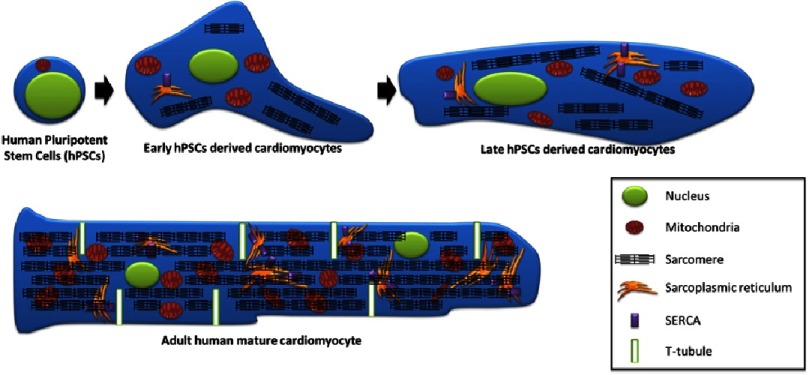
Schematic comparison of hPSCs, early and late hPSCs-CMs with adult mature CMs. Human pluripotent stem cells are small cells with a large nucleus/cytoplasm ratio. The cells possess limited sarcoplasmic reticulum and only few mitochondria. Early hPSCs-CMs express disorganized sarcomeric proteins. The morphology of the cells remains way smaller than adult cardiomyocytes and the lack of structure lead them to have more various morphology. The presence of SERCA on the surface of the sarcoplasmic reticulum allows them to display a transient calcium potential and a possible contraction. In late hPSCs-CMs the sacromeric proteins are more organized leading to have cells of more elongated shape. In parallel, the increase of the cytoplasm and mitochondria and sacoplasmic reticulum along with SERCA proteins ensure a more stable contractility of the cells. In contrast, adult cardiomyocytes present a well defined elongated shape where sacomeric proteins are highly organized. The cells are often multinucleated and there is a stron aboundance of mitochondria. More specifically, unlike early and late hPSC-CMs, T-tubule are present in adult cardiomyocytes that actively participate to the contractility of the cells. (Adapted from [6].)

Compared to adult CMs, PSCs-CMs are smaller, rounder in shape^26^, mostly single-nucleated^20^, always lack the t-tubules essential for excitation^19^ (as mentioned earlier), and their sacromeres lack M-bands (Figure 4).

*In vitro*, CMs derived from PSCs start expressing sarcomeric proteins as early as 6 days post induction, and a self-initiated contraction (beating) can be observed as early as 6-7 days post induction. In the following days, more and more cells will start beating and by day 10-12, most cells already display this self-initiated contractility^12^. This contractility can be maintained *in vitro* under appropriate conditions for months.

hPSC-CMs can be divided into two categories according to the time of their differentiation. Early-stage CMs (first two weeks post differentiation initiation) are cells which retain a proliferative capacity that resembles the embryonic or fetal CMs, while late-stage CMs (after more than three weeks post differentiation initiation) lose their ability to proliferate but acquire more mature characteristics^6,27,28^ (Figure 4).

### Proliferation, cell cycle arrest and ploidy

During fetal life, CMs proliferate rapidly but lose their proliferative ability as they withdraw from the cell cycle soon after birth^29^. Hence, adult CMs are considered to be one of the most slowly dividing cells^4^. As the proliferation halts, adult CMs undergo additional DNA synthesis and nuclear mitosis without the presence of the cytokinesis, which prevents the cells from dividing and results in multi-nucleation, or in some cases, extensive polyploidy of the CMs^30,31^.

Embryonic CMs exhibit a high expression of cyclins and cyclin-dependent kinases (Cdks) involved in the different phases of the cell cycle (G1, S, G2 and M), in addition to the proliferating cell nuclear antigen (PCNA) – a gene required for DNA replication^32^. The withdrawal of adult CMs from the cell cycle is due to the significant downregulation in the expression of the cyclins and Cdks after birth compared to levels in the fetal heart^32,33^. Proliferation of hPSCs-CMs is similar to the fetal CMs, and over time in culture their proliferative capacity decreases^26,34^.

Meis1, a transcriptional factor, has been also reported to be a crucial regulator of postnatal CMs proliferation as they activate CDK inhibitors p15, p16 and p21. It was shown *in vivo* that Meis1 expression increases from day 4, throughout adulthood, during neonatal heart development and regeneration. The deletion of Meis1 in mouse CMs resulted in the extension of postnatal CMs proliferation^35^.

Another cell cycle regulator is D2SV, which is a conserved variant of cyclin D2. D2SV was shown to induce the cell cycle exit of embryonic CMs by forming micro-aggregates that suppress the action of other cell cycle promoting proteins including CDK4, cyclin B1, and cyclin 2D, leading eventually to cell cycle exit^36^.

### Metabolism

Since the heart has a vital role of supplying the whole body with blood through its continuous contracting, a very high energy is required to maintain optimal function. CMs have a minimal storage of high-energy phosphate and the mitochondrion is responsible for more than 95% of the ATP used by the heart^37^. CMs have the highest contents of mitochondria among other cell types as they can occupy up to one third of the cell volume^38^. Terminally differentiated adult CMs have an increased mitochondrial oxidative metabolism due to the fatty acid β-oxidation, which is the major source for ATP synthesis in this stage. This eventually leads to the decrease in the proliferative capacity of the CMs^39^.

On the other hand, glycolysis is the major source of energy during the early development of CMs. This contributes to the proliferation of the embryonic CMs. As the CMs mature, they switch from glycolysis to mitochondrial oxidative metabolism. hPSCs-CMs rely mainly on glycolysis as a source for ATP synthesis^39^. In some other reports^40^, it has been shown that they use a mixture of glycolysis and oxidative metabolism as source of energy. We still don’t know whether long-term culture alters the hPSCs-CMs preferred energy substrate.

### Gene expression

On a molecular level, adult CMs express several specific markers including transcription factors, hormones, structural proteins and ion-channels^41^. Early cardiac-specific transcription factors are expressed like *GATA4*, *Nkx2.5*, *Isl-1*, *Tbx5* and *Mef2c* as well as cardiac structural proteins such as α-actinin (*ACTN*), sacromeric proteins, cardiac troponin T (*cTnT*), sarcomere myosin heavy chain (MHC) atrial and ventrical myosin light chains (*MLC-2A* and *MLC-2V*)^42^.

In hPSCs-CMs, the gene expression profile differs from that of pluripotent stem cells, mainly in the loss of pluripotency markers such as stage specific antigen 1 (SSEA1) and the up-regulation of mesodermal and cardiac markers including MESP1 and Nkx-2.5.

The previously mentioned, markers expressed by adult CMs are also expressed by the pluripotent stem cell-derived CMs^43^. More importantly however, the functional characteristics of hPSCs-CMs are similar to that of fetal heart tissue due to the lack of expression of functional genes including phospholamban (PLN) and calsequestrin^44,45^. PLN has a major role in regulating cardiac calcium signaling through the pumping of cytosolic calcium into the SR^46^ while calsedquestrin is a Ca^2+^ binding protein in the SR of the CMs^47^. The derived CMs also have an immature sarcoplasmic reticulum, which results in deregulation of the intracellular calcium handling^48^. The balance in MLC2A/MLC2V expression is more closely related to the proportion found in embryonic CMs than adult ones.

### Electrophysiology and calcium handling

CMs generate action potential through their ion channels. Calcium channels are essential to contractility function. Depolarization of the cardiac membrane leads to an influx of Ca^2+^ from the sarcoplasmic reticulum into the cardiac cell, which increases the myoplasmic free Ca^2+^ concentration^49^. This increase is transient, as the sodium-calcium exchanger moves the calcium back to the sarcoplasmic reticulum. The rise and fall in Ca^2+^ concentrations causes synchronous contraction of the CMs^50^. This finely adjusted calcium handling relies on various ion channels. Different types of ion channels are expressed during development, and the intensity of membrane potential is lower in embryonic CMs than mature adult ones (∼ −60 mV compared to ∼ −90 mV)^51^.

While most of the ionic channels are expressed in hPSCs-CMs, the level of expression differs from adult’s CMs and evolves along time in culture^52^. More interestingly, hPSCs-CMs were reported to display potassium channels considered to potentially be involved in arrhytmias^6,53,54^. As a consequence, many studies used hPSCs-CMs to study arrhytmia and for arrhythmia drug screening^55^. These arrhythmias seen to be more pronounced in early hPSCs-CMs than late hPSCs-CMs in accordance with the more mature profile of the latest^56,57^.

**Figure 5.**
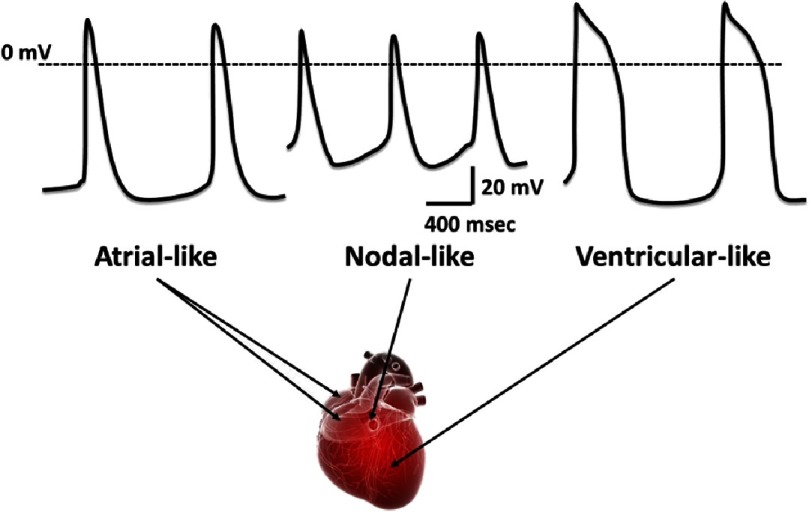
Schematic representation of the various action potential observed in different area of the heart. Cardiac differentiation from hPSCs leads to various type of cardiomyocytes whose action potential can differ. Most of the differentiation protocol leads to ventricular like cardiomyocytes with characteristic action potential. Though, some cells can display an action potential more specific to cardiomyocytes in the atrium, with a shorter plateau phase at the repolarization but of same intensity as ventricular cardiomyocytes. Finally a restricted number of cells present a nodal like action potential with small signals of restricted intensity but higher frequency.

**Figure 6.**
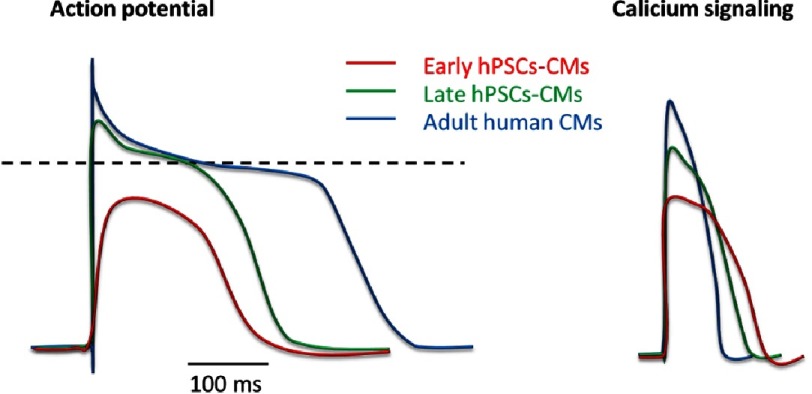
Approximate comparison of the action potential signal and calcium signal in early, late and adult cardiomyoctes. The action potential of hPSCs-CMs will grow both in intensity and only late hPSCs-CMs will display a plateau in the signal like adult cardiomyocytes. Similarly, the calcium signal of hPSCs-CMs will grow in intensity with time in culture but rarely reach the levels observed in adult CMs.

As previously mentioned, beating is observed from day 5-7 post differentiation initiation in hPSCs-CMs and can be maintained for months. The beat rate can vary broadly in hPSCs-CMs according to the cell line used, culture conditions and time in culture^28,58^. Different action potential witnessing heterogenic population (compared to adult CMs) are usually observed within the same differentiation plate with artrial, nodal and ventricular like action potentials (Figure 5). Action potential of early hPSCs-CMs usually range around –30 mV^59^, while late hPSCs-CMs can display action potential of ∼ −60 mV to –70 mV^28^ (Figure 6).

## Factors influencing maturation of hPSC derived cardiomyocytes

### Long term maintenance in culture:

The effect of long-term culturing of hPSCs-CMs’ maturation has been investigated. It was shown that upon culturing hPSCs-CMs up to one year, M-bands containing sacromeres were detected^60^. The appearance of M-bands is considered to be a distinctive feature of sarcomeric structural maturation of adult CMs^61,62^. Spontaneous beating can be detected in hPSCs-CMs as early as day 7 of differentiation and it was noticed that the beating rate of the cells in day 30 was significantly higher than that of day 360. hPSCs-CMs were considered immature because of the presence of cells that are double positive for MLCv and MLC2a which is one of the characteristics of fetal CMs^63^. Day 30 CMs had almost 56% of the cells double positive for the two myosin light chain proteins whereas the day 360 CM had only 36%. Ultrastructural analysis was performed on PSCs-CMs and they were classified according to the duration of culture into day 14, 30, 60, 90, 180 and 360. All the cells contained unaligned sacromeres lacking the M band that was only seen on day 360 along with the other A-, H-, I- and Z-bands. Day 360 cells had well-organized myofibrils and the amount of sarcomeric structure was increased compared to the other groups. In addition to that, the cell size of the hPSCs-CMs had increased significantly after the long term culture along with the number of multineucleated CMs. As for the functional characteristics, the long-term culturing of the cells led to an increase in the calcium release and uptake compared to cells in the early stages of differentiation. Electrophysiologically, long-term culturing of hPSCs-CMs resulted in increasing action potential amplitudes and faster upstroke velocities. All these data support the idea that hPSCs-CMs acquire a more mature phenotype after long term culture, but never acquire a fully mature profile nor display T-tubules^60^.

### Substrate stiffness

CMs’ morphology and function are highly dependent on the extracellular environment and matrix stiffness. Matrix elasticity was previously reported as essential to direct stem cell lineage specification^64^. More specifically, it was shown that hPSCs-CMs develop and mature better in substrates that are comparable with that of native tissue (myocardium)^65^. The contraction of CMs is regulated by the size of the calcium transient and the duration of the contraction (as explained before). The sarcoplasmic reticulum contains most of the calcium in adult CMs and is regulated by the SERCA2a and RyR2. SERCA2a is regulated by phospholamban (PLN), which is suppressed when it is phosphorylated^46^. In studies done on neonatal rat ventricular myocytes (NRVMs), cells were placed on collagen-coated polyacrylamide gel substrates with different elasticity modulus ranging from 1–50 kPa. The cells that were grown on a 10 kPa modulus (which is similar to native myocardium elasticity) showed the highest contractile force and calcium transient, in addition they exhibited well-defined sacromere and an increase expression of SERCA2a and RyR2 levels but no significant change was noticed on PLN levels. On the other hand, CMs grown on either softer or stiffer substrates showed less defined sacromeres^66^. This suggests that the plating of NRVNs on a stiffer substrate (compared to the native tissue) results in the less mature CMs with lower contractile force. In another study, they used polycarpolactone (PCL) for plating the cells with substrates of different densities (ranging from 1–133 Mpa Young’s moduli). The CMs that were plated on softer substrates showed more mature phenotype with well-defined sacromeres while the CMs grown on stiff PCL showed immature phenotype^67^.

### Cell alignment

An adult CM has a specific cell alignment that allows the electrical and mechanical coupling with the neighboring cells through contact junctions. When adult CMs are cultured, they start to die after sometime partially because of random orientation of the cells and lack of proper aligned cell to cell connections. Thus, scientists have tried to culture hPSCs-CMs on substrates that provide trophographical cues to regulate the cross talk between neighboring cells^68^. This significantly enhances the alignment of the cultured CMs and results in better conduction velocity. In general, compared to cells cultured randomly, aligned CMs exhibit properties more similar to the native heart.

### Electrical and mechanical stimulation

To have a normal cardiac function, electrical pulses are essential as they regulate the rhythm of CMs contraction. This is vital for maintaining the normal cellular structure and function of CMs. It was reported that when isolated adult CMs were cultured and subjected to continuous electrical pulsing, they show better contractile activity, sarcomere structure and intracellular structure compared to CMs cultured in the absence of electrical stimuli^69^. These changes in the sarcomeric organization are due to the up-regulation of structural proteins likes myosin heavy chain 7 (Myh7) and myosin light chain 2 (Myl2)^70^. Little work has been done in testing the effect of electrical stimulation on hPSC-CMs^48^, however it was reported that hESC-CMs subjected to electrical stimuli had a longer action potential duration and higher calcium influx and were more susceptible to caffeine^71^.

Mechanical stimulation or stretching is another broadly tested approach for CMs maturation. Acute stretch of CMs can activate the stretch-activated ion channels and hence allow the influx of Na+ and Ca2+ which eventually elicit action potentials. It was also reported that CMs stretching results in a more elongated phenotype^72^. Several studies showed that stretching of the substrate led to more mature profile of hESCs-CMs^73–76^. In addition, an increase was noticed in α-actinin, Nkx2.5 and GATA4 mRNA levels. Furthermore, the stretched CMs exhibited a synchronized beating while the un-stretched cells showed no synchronization in beating^77^.

### Chemical stimulation

#### Endothelin-1:

Endothelin-1 (ET-1) is a protein in humans encoded by the gene *EDN1*. It is produced by the vascular endothelial cells in response to many stimulants including: thrombin, angiotensin II, antidiuretic hormone and most importantly reactive oxygen species^78^.

Human embryonic CMs go under hypoxic stress during fetal development. This stimulates the expression of ET-1 which in turn contributes in causing the fetal CMs to prematurely exit the cell cycle. The CMs then increase in size (hypertrophy), their proliferation rates decrease and the DNA methylation result in binucleated cells. Naturally, this drove scientist to use ET-1 in vitro to increase hPSC-CMs maturation, and indeed, all together this indicates that ET-1 can drive maturation of CMs (hypertrophy, multinucleation, calcium handeling…). ET-1 was also found to influence strongly spontaneous Ca2+ oscillation *in vitro*^79^.

#### Triiodothyronine:

Thyroid hormones – especially triiodothyronine (T3) - play also an essential role in fetal CMs maturation by reducing their proliferative capacity towards the end of the gestational life^80^. It was shown that upon treatment with T3 for a week, hiPSCs-CMs have increased size and sacromere length. Additionally, the proliferation rate was slower and the cyclin-dependant kinase inhibitor levels were increased after treatment. The contractile force of the cells was enhanced and the rates of calcium release and uptake were also significantly increased. In addition to that, the mitochondrial respiratory capacity was increased after the T3 treatment^81^. All together these findings suggest that T3 can derive hPSCs-CMs maturation.

#### Neuregulin-1:

Neuregulin-1 is a protein encoded by the NRG1 gene and has multiple alternative splicing forms. It is involved in normal nervous system and cardiac development. In the heart, it acts as a cardioactive growth factor produced by cardiac microvascular endothelial cells (ECs) only of the endocardial vessels^82^. It has a vital role in the normal cardiac function of the adult heart. Neuregulins belong to the epidermal growth factor family. ErbB4 and ErbB2 are the main receptors for NRG-1 in the adult heart^83^ and upon binding it induces the formation of homodimers ErbB4/ErbB4 or heterodimers ErbB4/ErbB2^84^. The indispensable cardiac role of the NRG-1/ErbB signaling pathway was discovered as a side effect of trastuzumab treatment which is a monoclonal antibody directed against ErbB2 developed for breast cancer treatment. The antibody caused dilated cardiomyopathy and heart failure when given to patients with ErbB2 positive breast cancer^85^.

NRG-1/ErbB2 paracrine effect is highly important in the formation of the myocardial trabecula^86^. This was confirmed when tested with mice lacking neurogulin-1 or its receptors ErbB2 or ErbB4. The mice died during their mid-embryogenesis life because they lacked the myocardial trabeculae^87–89^. NRG-1 was also found to have a role in promoting the proliferation, hypertrophy and survival of cultured neonatal CMs^83^. It was shown that subsequent to ischemic heart failure, ECs increase the production of NRG-1, triggering all the previously described effects^90–92^. Recombinant human NRG-1 is now in phase 3 of clinic trials as adjuvant treatment following heart failure.

Altogether, this suggests that NRG-1 plays a crucial role in the development and the physiology of CMs. Similar effects on PSCs-CMs were investigated. Indeed, it was shown that the inhibition of NRG-1/ErbB signaling pathway during the differentiation of hESCs into CMs results in the increase in number of CMs with nodal-like phenotype^86,93^. A recent publication demonstrated that NRG-1 can not only successfully differentiated iPSCs into CMs but furthermore the produced CMs exhibited a more mature ventricular phenotype and mature electrophysiological properties after only 14 days^94^.

## Discussion

The past years have seen major advances in hPSCs-CMs differentiation, characterization and application. Despite the advances, *in vitro* generated CMs are only partially matured compared to adult CMs, which is holding back their use in cell transplantation, drug screening and disease modeling. Numerous treatment and cell culture systems have been tested to drive rapid maturation of the cells, but, to date, the long-term maintenance of cells in culture remains the foremost approach, with better morphological and functional maturation. Additionally, despite new techniques that brought more homogeneity, the maturity as well as the efficiency of differentiation depends heavily on the cell line. The beating speed, the action potential and the gene expression remain more efficient in hES- than in hIPS-derived CMs, and this should be taken into consideration in various research scenarios (disease modeling, drug screening or clinical application).

Despite this major limitation, and the note of caution brought by the publication of a non-human primate trial^7^, the enthusiasm generated by hPSCs potential for heart generation lead recently to the first embryonic stem cell trial for heart regeneration in human. In this trial, the selected cells defined as cardiac progenitors (CD15^+^ Isl-1^+^), by definition lack maturation steps and the major focus for the investigator was to avoid injection of undifferentiated cells (to avoid teratoma formation). Even if this represents a milestone in the clinical entry of hPSCs in heart disease treatment, the lack of maturity of the cells and possible consequences urges skepticism.

New approaches, combining combinations of the previously described maturation techniques could lead to more rapid and drastic maturation of the hPSCs-CMs. Delivery methods of the generated cells represents a large and active area of research. While intra muscular delivery of isolated cells remains the most widely-used approach, it has been shown that the engraftment efficiency of the CMs is very inefficient, and requires a huge number of cells to achieve tissue remuscularization^7,95^. To circumvent this hurdle, intensive work is performed on producing scaffolds where cells are pre-seeded before implantation. The materials employed, the cell seeding technique and the possibilities of using various maturation process discussed previously lead to the proposition that engineering hPSCs-CMs in tissues could drive more maturity than isolated cells when considered for clinical application and drug screening application^96^.
